# Integration of Ground- Penetrating Radar and Gamma-Ray Detectors for Nonintrusive Characterisation of Buried Radioactive Objects

**DOI:** 10.3390/s19122743

**Published:** 2019-06-18

**Authors:** Ikechukwu K. Ukaegbu, Kelum A. A. Gamage, Michael D. Aspinall

**Affiliations:** 1 Engineering Department, Lancaster University, Lancaster LA1 4YW, UK; m.d.aspinall@lancaster.ac.uk; 2 School of Engineering, University of Glasgow, Glasgow G12 8QQ, UK; Kelum.Gamage@glasgow.ac.uk

**Keywords:** ground-penetrating radar, gamma ray detector, sensor fusion, nuclear wastes, nuclear decommissioning, radiation detection, radiological characterisation

## Abstract

The characterisation of buried radioactive wastes is challenging because they are not readily accessible. Therefore, this study reports on the development of a method for integrating ground-penetrating radar (GPR) and gamma-ray detector measurements for nonintrusive characterisation of buried radioactive objects. The method makes use of the density relationship between soil permittivity models and the flux measured by gamma ray detectors to estimate the soil density, depth and radius of a disk-shaped buried radioactive object simultaneously. The method was validated using numerical simulations with experimentally-validated gamma-ray detector and GPR antenna models. The results showed that the method can simultaneously retrieve the soil density, depth and radius of disk-shaped radioactive objects buried in soil of varying conditions with a relative error of less than 10%. This result will enable the development of an integrated GPR and gamma ray detector tool for rapid characterisation of buried radioactive objects encountered during monitoring and decontamination of nuclear sites and facilities.

## 1. Introduction

The presence of radioactive objects in the shallow subsurface is a major public health risk because these objects can induce high levels of radiation above the ground. For example, a cobalt-60 source found buried at a depth of about 32 cm in a Cambodian hospital induced radiation levels of up to 60 mSv h^−1^ above the ground [1]. This is about 26,000-times the stipulated effective dose limit of 20 mSv per year [2]. Furthermore, chemical reactions in the soil can lead to the dissolution of these objects and subsequent contamination of groundwater. For example, the high energy penetrators used in ammunition are usually made from depleted uranium, which is a by-product of the nuclear fuel enrichment process. Many of these penetrators get lodged in the ground during military operations and become potential sources of groundwater contamination because of their high solubility in sand and other volcanic rock [3]. Therefore, it is important to promptly detect, and safely dispose these objects.

The first stage in the disposal of these buried radioactive objects is their characterisation. However, this process is challenging because of the difficulty in estimating the depth of these objects using traditional intrusive methods such as logging and core sampling [4,5]. Therefore, a number of nonintrusive depth estimation methods have been developed. These can be broadly divided into three categories, namely: empirical model methods; multiple photo peak methods; and shielding and collimator methods. The empirical model methods are based on establishing correlations between distinguishable features in part or all of the gamma spectrum and the depth of the buried radioisotope. They include: peak-to-valley ratio [6,7], peak-to-scatter ratio [8,9], principal component analysis [10,11,12], and machine learning [5,13,14] methods. However, these methods result in models whose parameters typically have no physical significance. Furthermore, the use of machine learning requires a significant amount of data for training. The multiple photo peak methods [15,16] exploit the difference in the attenuation of two energy peaks in the gamma spectrum in order to estimate the depth of the source. Consequently, they are limited to radioisotopes with two or more photo peaks that are sufficiently separated in the gamma spectrum.

The shielding and collimator methods [17,18,19] use different shielding and collimator configurations to obtain multiple measurements from which the depth of the radioactive source can be estimated. These methods have been shown to yield more accurate results compared to other methods [17] and can be used with any radioisotope. However, the required multiple measurements can only be acquired sequentially. This can significantly increase the data acquisition time because the acquisition of the spectrum of a buried source usually requires a long dwell time due to significant attenuation. In addition, in order to limit the minimum number of measurements required to estimate the depth to only two, the value of the bulk density of the soil is typically assumed to be known. However, the bulk density of soil depends on the current condition of the soil, and this varies from one location to another. Therefore, assuming a constant or generic value will result in errors in the estimated quantities. Furthermore, the use of historical values will not account for the changes in the soil density that would have occurred over time due to environmental factors such as rain fall and temperature changes.

Therefore, this work presents the development of a method for integrating gamma-ray detectors and ground-penetrating radar (GPR) for the retrieval of the soil density, depth and radius of a buried radioactive object. This eliminates the need for the soil density value to be known a priori. The method also used two horizontally-separated detectors to enable simultaneous acquisition of the required measurements, thereby solving the problem of sequential data acquisition. This will improve the rapid characterisation of buried radioactive wastes.

## 2. Theoretical Framework

**Figure 1:**
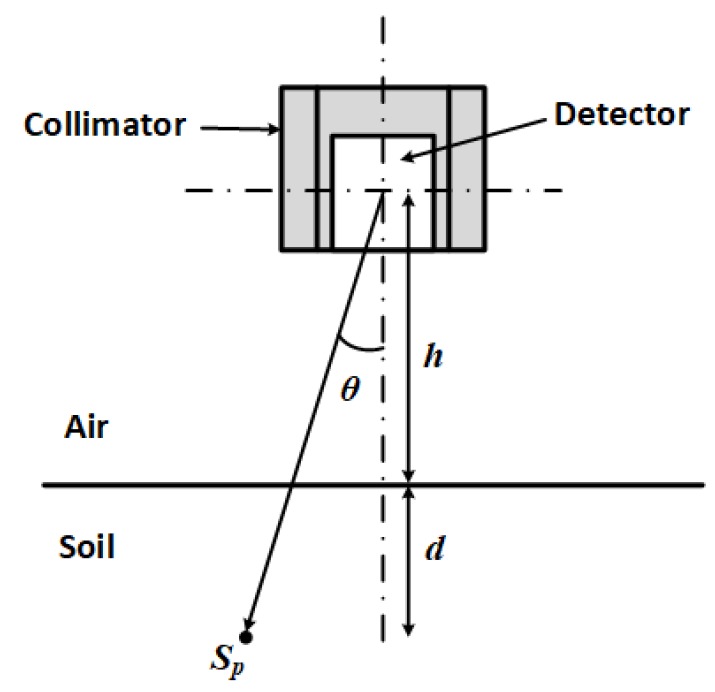
Geometry and parameters for estimating the flux (measured by the detector) due to the point source $S_{p}$ in the soil.

For a radioactive point source buried in an air-soil half-space as shown in Figure 1, the flux $F_{p}$ measured by the detector placed above the ground is given by [20]:

(1) $$F_{p}=\frac{S_{p}A_{r}\left(E,\theta \right)C_{e}\left(E\right)}{4\pi {\left(\frac{h+d}{\cos\theta }\right)}^{2}}e^{-\mu_{m}\left(E\right)\rho_{a}\frac{h}{\cos\theta }}e^{-\mu_{m}\left(E\right)\rho_{b}\frac{d}{\cos\theta }}$$

where *E* is the energy of the point source (keV), θ is the angle of incidence of the source with the detector (radians), *d* is the depth of the source in the soil (cm), $S_{p}$ is the activity of the source (Bq) and $A_{r}\left(E,\theta \right)$ is the angular response of the detector to a point source of energy *E* incident at angle θ. This is a dimensionless quantity and is obtained by measuring the response of the detector to a point source at angles varying from 0–π/2. This calibration should be done with the collimator in place if the detector is to be used with a collimator. $C_{e}\left(E\right)$ is the detector’s centreline efficiency (cps cm^2^ Bq^−1^) and is calculated from the flux due to a source of known activity placed at a known distance *z* along the centerline, i.e.,:

(2) $$C_{e}=\frac{F_{p}4\pi z^{2}}{S_{p}}$$

where $\mu_{m}$ is the mass attenuation coefficient of the point source at energy *E* (cm^2^ g^−1^), $\rho_{a}$ is the density of air (g cm^−3^), *h* is the distance from the ground surface to the centre of the detector and $\rho_{b}$ is the bulk density of soil (g cm^−3^).

If the buried object is assumed to be disk-shaped and the contamination is at most 1–2 mm below the object’s surface, then it can be approximated as a planar disk source, and the flux $F_{a}$ measured by the detector is obtained by integrating Equation (1) over the area of the disk, i.e.,:

(3) $$F_{a}=\int_{0}^{2\pi }\int_{0}^{r}\;\frac{S_{a}A_{r}\left(E,\theta \right)C_{e}\left(E\right)}{4\pi {\left(\frac{h+d}{\cos\theta }\right)}^{2}}e^{-\mu_{m}\left(E\right)\rho_{a}\frac{h}{\cos\theta }}e^{-\mu_{m}\left(E\right)\rho_{b}\frac{d}{\cos\theta }}\;r\;drd\phi$$

where *r* and ϕ are the radius (cm) and angle (radians) of the disk source in polar coordinates and $S_{a}$ is activity per unit area (Bq cm^−2^).

In most buried radioactive source surveys, the quantities of interest are the activity and depth of the source of the radiation; both of which are estimated from the ratio of two measurements [19]. In other words, the ratio of two measured fluxes $F_{1}$ and $F_{2}$ acquired using different detector configurations is a function that depends only on the source depth, i.e.,

(4) $$\frac{F_{2}}{F_{1}}=ratio\left(d\right)$$

The depth estimated from Equation (4) can then be used to estimate the source activity using Equation (1) or (3) for a point or planar source. However, this two-measurement procedure assumes that the bulk density of the soil is known. This requirement can be eliminated by acquiring a third measurement [19]; however, this will increase the data acquisition time.

**Figure 2:**
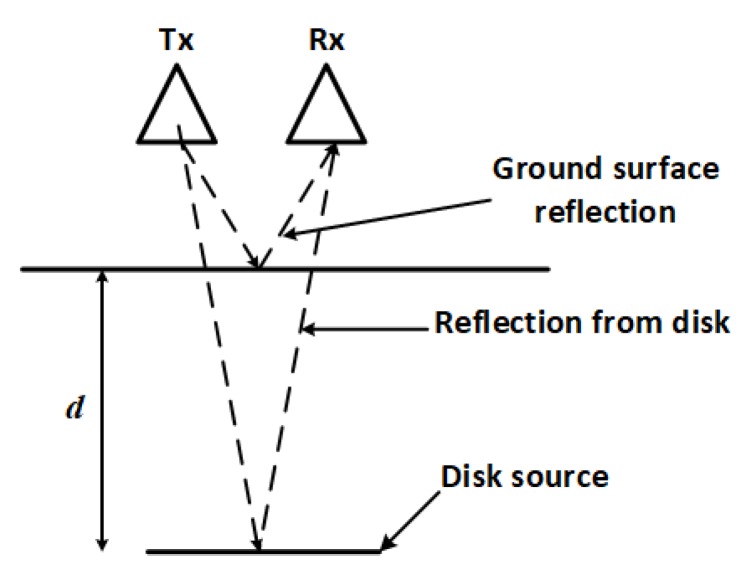
Operation of a ground-penetrating radar (GPR) system. Signals from the transmitter (Tx) are reflected by objects and detected by the receiver (Rx).

GPR has the potential of solving this density-dependency dilemma. A GPR system operates by sending electromagnetic signals into the ground and measuring any portion of the signal that is reflected by interfaces or objects in the signal propagation path. Using the illustration in Figure 2, the time *t* between the reception of the reflection from the ground and that from the disk source is given by:

(5) $$t=\frac{2d}{v}=\frac{2d}{\frac{c}{\sqrt{\epsilon_{b}}}}$$

where *v* is the speed of the signal in the soil (m s^−1^), *c* is the speed of light (299,792,458 m s^−1^) and $\epsilon_{b}$ is the relative bulk permittivity of the soil (unitless). It should be noted that Equation (5) assumes that both the transmitting (Tx) and receiving (Rx) antennas are close to each other. Porous materials such as soil can be considered as a three-phase mixture of air, water and solid particles [21]. Therefore, their bulk permittivity is a function of the permittivities of these phases and their proportional composition in the material. Various formulas have been proposed to express this relationship; however, in a comparative study [22], it was shown that the formula based on the exponential mixing rule [21] with the exponent value of 0.65 gave the best result across a variety of materials. This formula is given by:

(6) $$\epsilon_{b}^{0.65}=\left(\frac{\rho_{b}-W_{c}}{\rho_{s}}\right)\epsilon_{s}^{0.65}+\left(1-\frac{\rho_{b}-W_{c}}{\rho_{s}}-W_{c}\right)\epsilon_{a}^{0.65}+W_{c}\epsilon_{w}^{0.65}$$

where the exponent value of 0.65 was obtained from the work of Dobson et al. [23], $\rho_{s}=2.65$ g cm^−3^ is the solid particle density for soils, $W_{c}$ is the volumetric water content (%), $\epsilon_{s}=4.7$ is the solid particle relative permittivity for soils [23,24], $\epsilon_{a}=1$ is the relative permittivity of air and $\epsilon_{w}$ is the relative permittivity of water, which is given by the real part of the modified Debye’s equation [24], i.e.,

(7) $$\epsilon_{w}=\epsilon_{w,\infty }+\frac{\epsilon_{w,0}-\epsilon_{w,\infty }}{1+{\left(2\pi f\tau_{w}\right)}^{2}}$$

where $\epsilon_{w,\infty }=4.9$ is the relative permittivity of water at infinity, $\epsilon_{w,0}$ is the static relative permittivity of water, *f* is the frequency of the GPR (Hz) and $\tau_{w}$ is the water relaxation time (s). Both $\epsilon_{w,0}$ and $\tau_{w}$ depend on temperature *T* (${}^{\circ }$C) and are given by Equations (8) and (9), respectively [25,26].

(8) $$\epsilon_{w,0}=88.045-0.4147\times T+6.295\times 10^{-4}\times T^{2}+1.075\times 10^{-5}\times T^{3}$$

(9) $$\tau_{w}=\frac{1}{2\pi }\left(1.1109\times 10^{-10}-3.824\times 10^{-12}\times T+6.938\times 10^{-14}\times T^{2}-5.096\times 10^{-16}\times T^{3}\right)$$

Combining Equations (5) and (6) will yield Equation (10), which can be solved simultaneously with Equation (4) to estimate both the soil bulk density and the depth of the source. This integration of the data from the GPR and gamma detectors can be considered as a type of low-level multisensor data fusion where data from different sensors are combined using physical models to enable or improve the estimation of physical parameters [27].

(10) $${\left(\frac{2d}{ct}\right)}^{1.3}=\left(\frac{\rho_{b}-W_{c}}{\rho_{s}}\right)\epsilon_{s}^{0.65}+\left(1-\frac{\rho_{b}-W_{c}}{\rho_{s}}-W_{c}\right)\epsilon_{a}^{0.65}+W_{c}\epsilon_{w}^{0.65}$$

**Figure 3:**
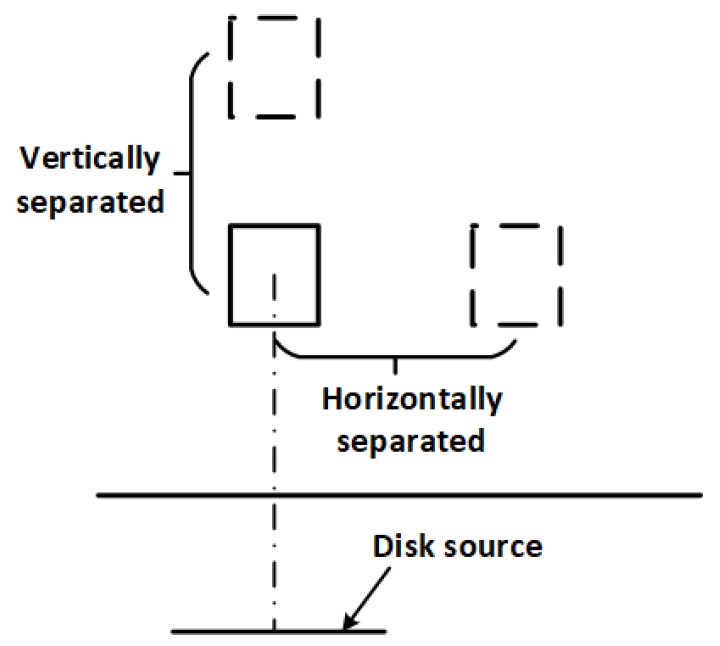
Two ways of arranging two detectors to measure the flux from the disk source. The horizontally-separated arrangement allows both fluxes to be measured simultaneously because none of the detectors is obstructed.

Another important consideration is how to arrange the sensors (i.e., the gamma detectors and GPR antenna) for efficient data acquisition. Preferably, the arrangement should be such that the sensors can operate simultaneously. Two ways of positioning two gamma detectors for the measurement of the radiation fluxes are illustrated in Figure 3. In the first arrangement, both detectors are vertically displaced by a fixed distance. However, this configuration makes it difficult to simultaneously measure the fluxes from both detectors because the field of view of the upper detector is completely or significantly occluded by the lower detector for small objects. This problem does not occur in the second arrangement where the second detector is horizontally displaced from the reference detector. This arrangement also has the additional advantage of allowing the GPR antenna to be mounted between both gamma detectors thereby creating a more compact sensor arrangement. However, the calculation of the angle of incidence (θ in Equation (3)) for the second detector needs to be modified to account for the horizontal separation. The modified expression is given by;

(11) $$\begin{array}{c} \theta =\arctan\left(\frac{a}{h+d}\right)\\ \mathrm{where}\;a=\sqrt{{\left(x+r\cos\phi \right)}^{2}+{\left(r\sin\phi \right)}^{2}}\\ \mathrm{and}\;x\;\mathrm{is}\;\mathrm{the}\;\mathrm{horizontal}\;\mathrm{separation}. \end{array}$$

## 3. Materials and Methods

The numerical modelling and simulation tools used were Monte Carlo N-Particle Version 5 (MCNP5) [28] and gprMax Version 3.1.4 [29]. MCNP5 is a collection of software codes that is used to simulate the transportation of subatomic particles, e.g., gammas, neutrons, etc., and their interaction with materials using Monte Carlo statistical techniques. It is widely used in the modelling and analysis of nuclear radiation structures and systems and has been extensively proven to have good agreement with experimental results. gprMax is an open source software code used to simulate the propagation of GPR signals. At its core, gprMax is a finite-difference time-domain electromagnetic wave solver that uses Yee’s algorithm to solve the three-dimensional Maxwell’s equations. Its results have also been extensively validated with experiments [30].

### 3.1. Selection and Modelling of Sensors

**Figure 4:**
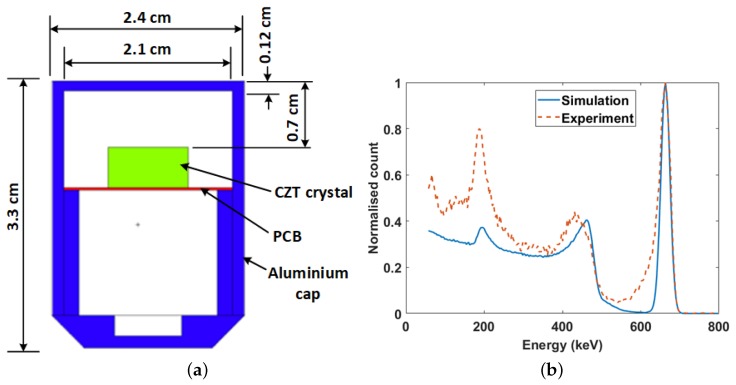
(**a**) MCNP5 model of the gamma detector. The crystal volume is 1 cm × 1 cm × 0.5 cm; (**b**) Experimental and simulated Cs-137 spectrum from the model and real detector.

The gamma detector used in the study was the CZT/500S from Ritec (Riga, Latvia). It is a hemispherical cadmium zinc telluride (CZT) semiconductor detector with a sensitive volume of 0.5 cm^3^ (Figure 4a). The detector was chosen because of its size and good spectroscopic properties. In addition, unlike high purity germanium (HPGe) detectors, CZT detectors do not require a cooling system; therefore, they are very portable and easy to integrate with other systems. Figure 4b shows the simulated and experimental Cs-137 spectrum from the model and real detectors, respectively. A very good alignment of the spectrum key features can be observed. The tailing effect in the Compton valley of the spectrum from the experiment was due to incomplete charge collection caused by poor electron-hole mobility. This is a characteristic feature of CZT detectors. This feature was not modelled because of the additional complexity required. However, this will not affect the results of the study because the ratio of the area under the photo peak for two simulated spectra will be the same as that for two experimental spectra. The difference in the position of the Compton peak was likely due to nonlinearity in the real detector, while the higher background below 300 keV in the spectrum from the experiment can be attributed to backscatter from surrounding objects.

**Figure 5:**
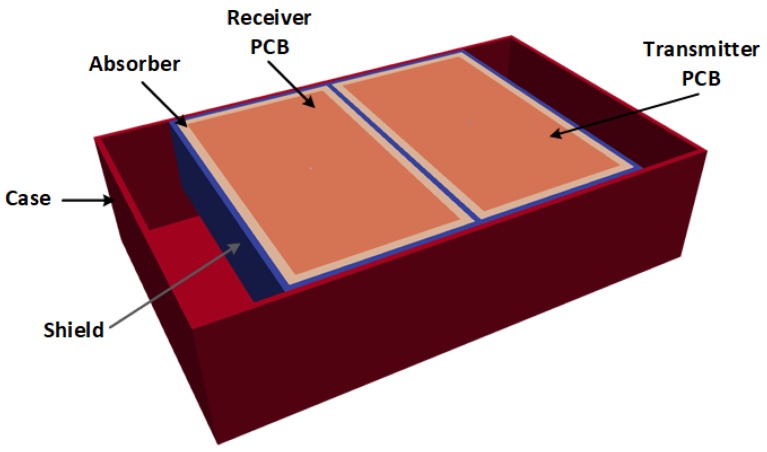
gprMax model of the 1.5-GHz antenna from GSSI Inc. The antenna dimensions are 17 cm × 10.8 cm × 4.3 cm (L×W×H). The skid plate underneath the casing has been removed to show the inside of the antenna.

The selected GPR antenna for the study was the 1.5-GHz antenna (Model 5100) from GSSI Inc. (Nashua, NH, USA). The gprMax model of this antenna is shown in Figure 5. The antenna consists of a pair of transmitter and receiver bow-tie antennas printed on a circuit board. The antennas are surrounded by microwave absorbers, which in turn are surrounded by a metallic shield. The entire assemble is enclosed in a polypropylene case. The development and experimental validation of the model can be found in [30,31]. It should be noted that the actual centre frequency of the antenna model was 1.71 GHz with a fractional bandwidth of 103%.

### 3.2. Measurement Scenario Modelling

**Figure 6:**
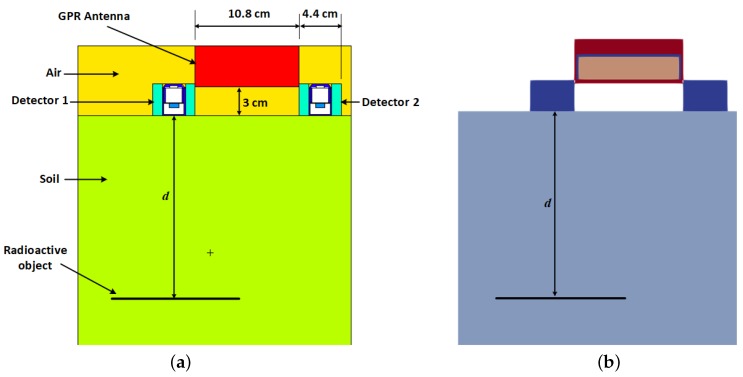
Model of the measurement scenario. The radioactive object is a metallic disk with Cs-137 radioactive contamination. (**a**) MCNP5 model of the measurement scenario. The gamma detectors are surrounded by 1 cm-thick lead collimators with an inner radius of 2.4 cm and height of 3.3 cm; (**b**) gprMax model of the measurement scenario. All labels and dimensions are the same as (**a**).

The measurement scenario was modelled both in MCNP5 and gprMax. The MCNP5 model of the measurement scenario is shown in Figure 6a. The radioactive object was modelled as a planar disk source with uniform activity. This is typical of stainless steel objects whose surfaces become activated by neutron flux in nuclear reactors [32]. The radioisotope used was Cs-137 with a photo peak energy of 662 keV. Each gamma-ray detector was placed in a cylindrical collimator with inner radius, thickness and height of 2.4 cm, 1.0 cm and 3.3 cm, respectively. The collimator was modelled as an alloy of tungsten (95% W, 3.5% Ni and 1.5% Fe) with a density of 18 g cm^−3^ [33]. The horizontal distance between the gamma detectors was selected such that it can fit the width of the GPR antenna. The antenna was modelled as a propylene box since it was not an active component in the MCNP5 simulation. The soil used in the model was a typical soil (51.4% O, 0.6% Na, 1.3% Mg, 6.8% Al, 27% Si, 1.4% K, 5.1% Ca, 0.5% Ti, 0.07% Mn and 5.6% Fe) with a dry density of 1.52 g cm^−3^ [34].

The gprMax model of the measurement scenario is shown in Figure 6b. This is a replication of the MCNP5 model using the gprMax antenna model described in Section 3.1. The detectors were modelled as metallic cylinders since only the lead collimator part of the gamma detectors will affect the GPR signals. The radioactive object was modelled as a metallic disk of thickness 0.5 cm. The two properties required to replicate the soil in gprMax were the bulk permittivity and the bulk conductivity. The bulk permittivity was calculated using Equations (6)–(9) at a temperature of 20 ${}^{\circ }$C. The bulk conductivity was calculated using [35]:

(12) $$\sigma_{b}=\frac{\sigma_{w}\left(\epsilon_{b}-4.1\right)}{\epsilon_{w}}$$

where $\sigma_{b}$ is the soil bulk conductivity (Sm^−1^) and $\sigma_{w}$ is the conductivity of pore water (0.05 Sm^−1^ [36]).

### 3.3. Simulation and Data Processing

Two sets of simulations were performed: MCNP5 simulations to measure the gamma fluxes due to the buried radioactive object and gprMax simulations to measure the time of flight (signal travel time) of the GPR signal to the buried radioactive object.

In the MCNP5 simulations, disk sources of radii of 3 cm, 9 cm and 15 cm were separately buried in the soil at depths varying from 12 cm–28 cm at 4-cm intervals. All the activities of the sources were normalised to 1 Bq cm^−2^, unless otherwise stated. After simulation, a Gaussian function was fitted to the spectra from the gamma ray detectors in order to estimate the number of full energy photons detected. This is the required flux due to the buried radioactive object. The energy range used for the estimation was from 655–672 keV.

**Figure 7:**
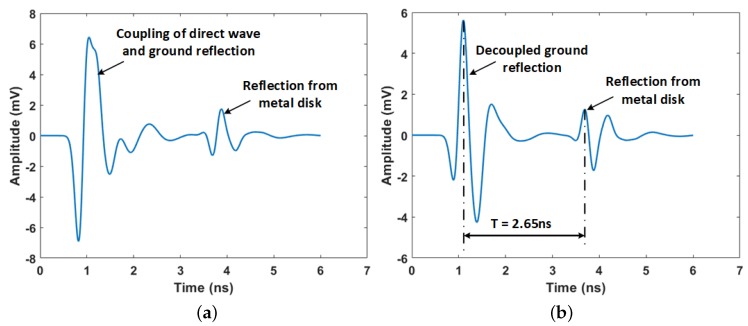
GPR signal for metal disk of a radius of 3 cm buried at 24 cm in dry soil, (**a**) Raw GPR signal with coupled direct wave and ground reflection; (**b**) GPR signal after subtraction of the GPR antenna’s system response.

In the gprMax simulations, the radioactive object was also buried in the soil at depths varying from 12 cm–28 cm at 4-cm intervals. The GPR signal was then transmitted and the reflected signals recorded for processing. The first step in processing the GPR data was the subtraction of the antenna’s system response from that acquired from the measurement scenario. The antenna’s systems response is the measured response when the antenna is in air or free space. This subtraction process decoupled the reflection due to the ground surface from the direct signal from the transmitter to the receiver. This made the reflected signal from the ground surface easily identified. The required signal travel time was then the time between the ground reflection and the reflection due to the metallic disk. This process is illustrated in Figure 7a,b.

Using the estimated gamma fluxes and the signal travel times, Equations (4) and (10) were simultaneously solved to obtain the soil density, depth and radius of the buried radioactive object. These results are presented and discussed in the following section.

## 4. Results and Discussion

**Figure 8:**
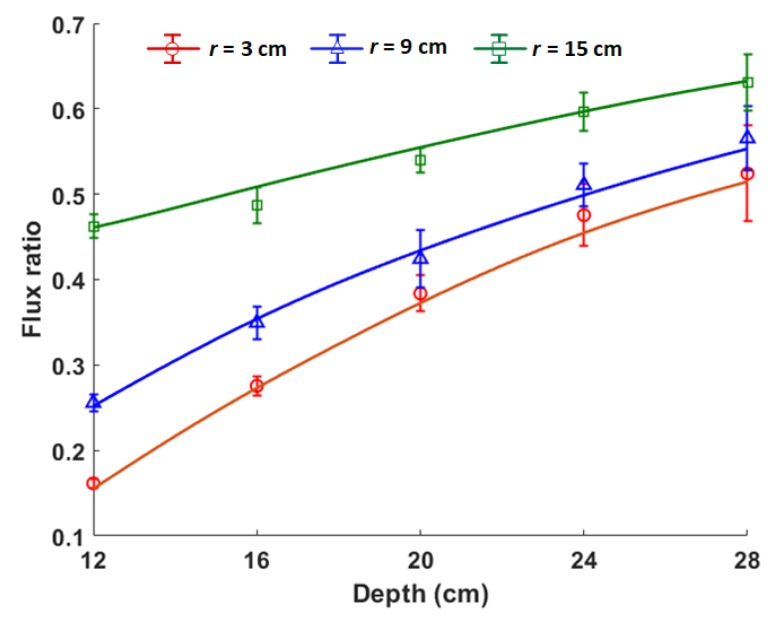
Flux ratio (i.e., $F_{2}/F_{1})$ for sources of radii of 3 cm, 9 cm and 15 cm buried at various depths in dry soil ($\rho_{b}=1.52$ g cm^−3^). The solid lines are calculated values, while the markers are the values from the simulation.

The calculated (solid lines) and simulated (markers) ratios of the fluxes (i.e., Equation (4)) from the gamma detectors for disk sources of different radii buried at different depths in the dry soil are shown in Figure 8. The uncertainty in the flux ratio was calculated using Equation (13), where $\delta F_{1}$ and $\delta F_{2}$ are the uncertainties in the fluxes from Detectors 1 and 2 as calculated by MCNP5. A decreasing dependency of the ratios on depth can be observed as the depth increased. This is indicated by the plateauing of the curves and the increasing error bars as the depth increased. This is caused by the exponential attenuation of the gamma rays as the depth of the source increased. This effect can be mitigated in practice by increasing the measurement time or by using a detector with higher efficiency. A decrease in the dependency of the ratios on depth can also be observed as the source radius increases. This is because the part of the source in the field of view of Detector 2 increases as its radius increases. Therefore, its measured flux will become increasingly the same as that measured by Detector 1 since the source has uniform activity.

(13) $$\mathrm{Flux}\;\mathrm{ratio}\;\mathrm{uncertainty}=\left|\frac{F_{2}}{F_{1}}\right|\sqrt{{\left(\frac{\delta F_{2}}{F_{2}}\right)}^{2}+{\left(\frac{\delta F_{1}}{F_{1}}\right)}^{2}}$$

The depths and densities obtained by simultaneously solving Equations (4) and (10) using the flux ratios in Figure 8 and the signal travel time from GPR measurements are shown in Table 1. The values in parentheses are the relative error in percentage. It can be observed that the estimated depths are within 5% of their actual values while most of the estimated densities are within 9% of their actual values. The density estimates with high errors are those obtained when the sources were buried at 12 cm. This is likely caused by the fact that the sources have a large incident angle with respect to Detector 2 when buried at shallow depths. This results in the reduction of the geometric efficiency of Detector 2.

Table 2 shows the depth and density estimates for a disk source (3 Bq cm^−2^) of a radius of 3 cm buried at a depth of 20 cm in soil of different densities and and volumetric water contents. The estimates in the first row were obtained using the proposed integrated GPR and gamma ray detectors approach. The values in the second row were obtained using the measurements from only the two gamma-ray detectors by minimising the following function:

(14) $$\mathrm{minimise}:\;\frac{{\left(R_{calc}-R_{sim}\right)}^{2}}{R_{sim}}$$

where $R_{calc}$ and $R_{sim}$ are the calculated and simulated flux ratios respectively. It can be observed that the combination of the gamma detector and GPR measurements significantly improved the depth and density estimates especially at high densities and water contents. This is because the additional measurement from the GPR constrained the solution space to the correct values. The solution space can also be constrained by using a third gamma detector measurement; however, this will either increase the data acquisition time if the measurements are acquired sequentially or require the design of a complicated measurement geometry for simultaneous measurement of all three fluxes. Conversely, this GPR integration approach is fast, simple, and produces good results.

Finally, the results presented so far assumed that the size (i.e., radius) of the disk source is known. However, this is typically not the case in practice. Therefore, the retrieval of the radius of the disk source was also investigated. Since this would require the estimation of three unknowns using two equations, the problem was reformulated as a constrained minimisation problem where Equations (10) and (7) are the objective and constraint functions, respectively. The result for disk sources of different radii buried in the soil at a depth of 12 cm is shown in Table 3. Good estimates can be observed as all of the estimated values had relative errors of less than 10% except the density and radius estimates for the disk source of radius of 3 cm. This large error in the estimates for the disk source of radius 3 cm is likely due to the large incident angle for Detector 2 at shallow depths, which reduced the number of gamma rays reaching the detector. This reduction in the flux measured by Detector 2 at shallow depths is more pronounced if the radius of the disk source is small. However, the results confirmed the ability of the integrated gamma detector and GPR method to estimate the key parameters of soil density, depth and radius of buried disk sources, simultaneously. Furthermore, this technique can also be used with other radioisotopes (e.g., Co-60) by substituting the mass attenuation coefficient at the photo peak energy of the radioisotope in Equation (3).

## 5. Conclusions

The integration of gamma detectors and GPR for nonintrusive characterisation of buried radioactive objects has been presented. The results showed that this integrated approach is able to retrieve the key parameters of soil density, depth and radius of disk-shaped radioactive objects buried in soil of varying conditions simultaneously. It also showed that by using two horizontally-separated gamma detectors, all the measurements required for the estimation process can be acquired simultaneously, thereby reducing the time associated with sequential data acquisition. However, the method is currently limited to objects having surface radioactive contamination that can be approximated by a disk. Therefore, there is a need to develop the method further to account for objects of different shapes. Finally, this study will form the basis for the development an integrated gamma detector and GPR system. Such a system will enable the rapid characterisation of buried wastes encountered during the decommissioning of nuclear sites and facilities.

## Figures and Tables

**Table 1:** Simultaneously-estimated depths and soil densities for disk sources of different radii buried at different depths in dry soil. The values in parentheses are the relative error in percentage.

Actual Values		Estimated Values					
		*r* = 3 cm		*r* = 9 cm		*r* = 15 cm	
d(cm)	$\rho_{b}$ (g cm^−3^)	d (cm)	$\rho_{b}$ (g cm^−3^)	d (cm)	$\rho_{b}$ (g cm^−3^)	d (cm)	$\rho_{b}$ (g cm^−3^)
12	1.52	11.8 (2)	1.36 (11)	11.9 (1)	1.34 (12)	12.2 (1)	1.25 (18)
16	1.52	15.7 (2)	1.42 (7)	15.7 (2)	1.43 (6)	15.2 (5)	1.54 (1)
20	1.52	19.8 (1)	1.41 (7)	19.6 (2)	1.45 (5)	19.0 (5)	1.57 (3)
24	1.52	24.0 (0)	1.38 (9)	23.1 (4)	1.52 (0)	23.5 (2)	1.46 (4)
28	1.52	27.7 (1)	1.43 (6)	27.9 (0)	1.41 (7)	27.3 (2)	1.48 (3)

**Table 2:** Depth and density estimates for a disk source of radius 3 cm buried at a depth of 20 cm in three different soil conditions. The values in parentheses are the relative error in percentage.

Estimation Method	Soil 1 ($\rho_{b}=1.67$ g cm^−3^,		Soil 2 ($\rho_{b}=1.82$ g cm^−3^,		Soil 3 ($\rho_{b}=1.97$ g cm^−3^,	
	$W_{c}=15\%$)		$W_{c}=30\%$)		$W_{c}=45\%$)	
	*d* (cm)	$\rho_{b}$ (g cm^−3^)	*d* (cm)	$\rho_{b}$ (g cm^−3^)	*d* (cm)	$\rho_{b}$ (g cm^−3^)
gamma detector and GPR	19.8 (1)	1.61 (4)	19.7 (2)	1.93 (6)	19.8 (1)	2.12 (8)
gamma detector only	19.17 (4)	1.48 (11)	17.6 (12)	1.5 (18)	16.83 (16)	1.5 (18)

**Table 3:** Estimated depths, densities and radii values for disk sources of varying radii buried in the dry soil at a fixed depth of 12 cm. The values in parentheses are the relative error in percentage.

Actual Values			Estimated Values		
d (cm)	$\rho_{b}$ (g cm^−3^)	r (cm)	d (cm)	$\rho_{b}$ (g cm^−3^)	r (cm)
12	1.52	3	10.9 (9)	1.64 (8)	6.6 (120)
12	1.52	9	11.5 (4)	1.47 (3)	9.6 (7)
12	1.52	15	11.6 (3)	1.43 (6)	15.1 (1)
